# Introduction of a modified double-lumen tube

**DOI:** 10.1007/s00540-018-2511-y

**Published:** 2018-05-22

**Authors:** Shinsuke Hamaguchi

**Affiliations:** Department of Anesthesiology and Pain Medicine, Dokkyo University School of Medicine, 880 Kitakobayashi, Mibu, Tochigi 321-0293 Japan

**Keywords:** Double-lumen tube, Bronchial cuff, Deflation

To the Editor:

Since 1949, the double-lumen tube (DLT) has played an important role in safe anesthetic management for intrathoracic surgeries, such as lung or esophageal surgeries requiring differential lung ventilation (DLV) [1, 2]. Here, I present a modified DLT to safely perform DLV.

There was a clinical problem, where Broncho-Cath™ (Covidien, USA) could not deflate bronchial cuff (blue cuff) when the tube was bent to a certain shape. The defective bronchial cuff deflation might be attributed to the kink in intratubal bronchial cuff lumen due to the curvature of it (supplementary Fig. 1).

Therefore, the location of bronchial cuff vent intratubal lumen in the existing tube may be inadequate. I discussed this aspect with the staff of Covidien Japan and suggested changes to the position of the cuff air delivery lumen while maintaining the strength and elasticity of the tube. Specifically, the modified Broncho-Cath™ exhibited three intratubal lumens in a position that is difficult to kink, which differs from the previous two intratubal lumens type (supplementary Fig. 2). After customization by the staff of Covidien Japan based on my opinion, the tube has been marketed as the Shiley™ Endobronchial Tube.

## Electronic supplementary material

Below is the link to the electronic supplementary material.

Figure 1 Previous type of double-lumen tube. **a** Bronchial cuff lumen, **b** tracheal cuff lumen (X-ray opaque line), and **c** kink of the bronchial cuff lumen may occur in this line (PPTX 588 KB)

Figure 2 Modified double-lumen tube. **a** Bronchial cuff lumen, **b** tracheal cuff lumen (X-ray opaque line), **c** tracheal cuff lumen, **d** no lumen (X-ray opaque line) (PPTX 1038 KB)
