# Supplementary figures and images for: Introduction of a modified double-lumen tube
Source: J Anesth. 2018 May 22;32(4):652. doi: 10.1007/s00540-018-2511-y (PMC6096719; doi:10.1007/s00540-018-2511-y)

## Slide 1
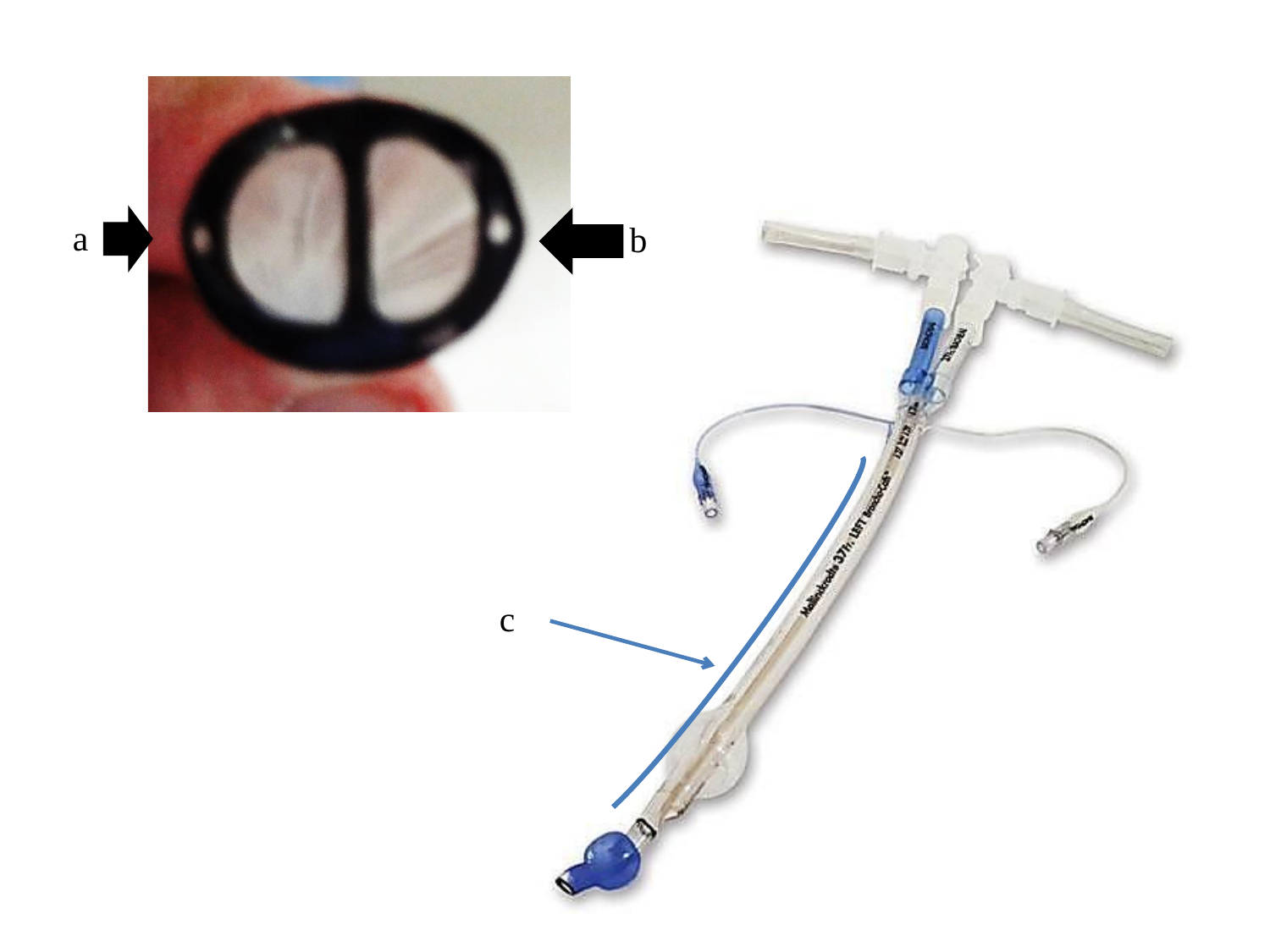

a
b
c

Supplement: Supplementary file 1 — Figure 1 Previous type of double-lumen tube. a Bronchial cuff lumen, b tracheal cuff lumen (X-ray opaque line), and c kink of the bronchial cuff lumen may occur in this line (PPTX 588 KB) [file 540_2018_2511_MOESM1_ESM.pptx]

## Slide 1
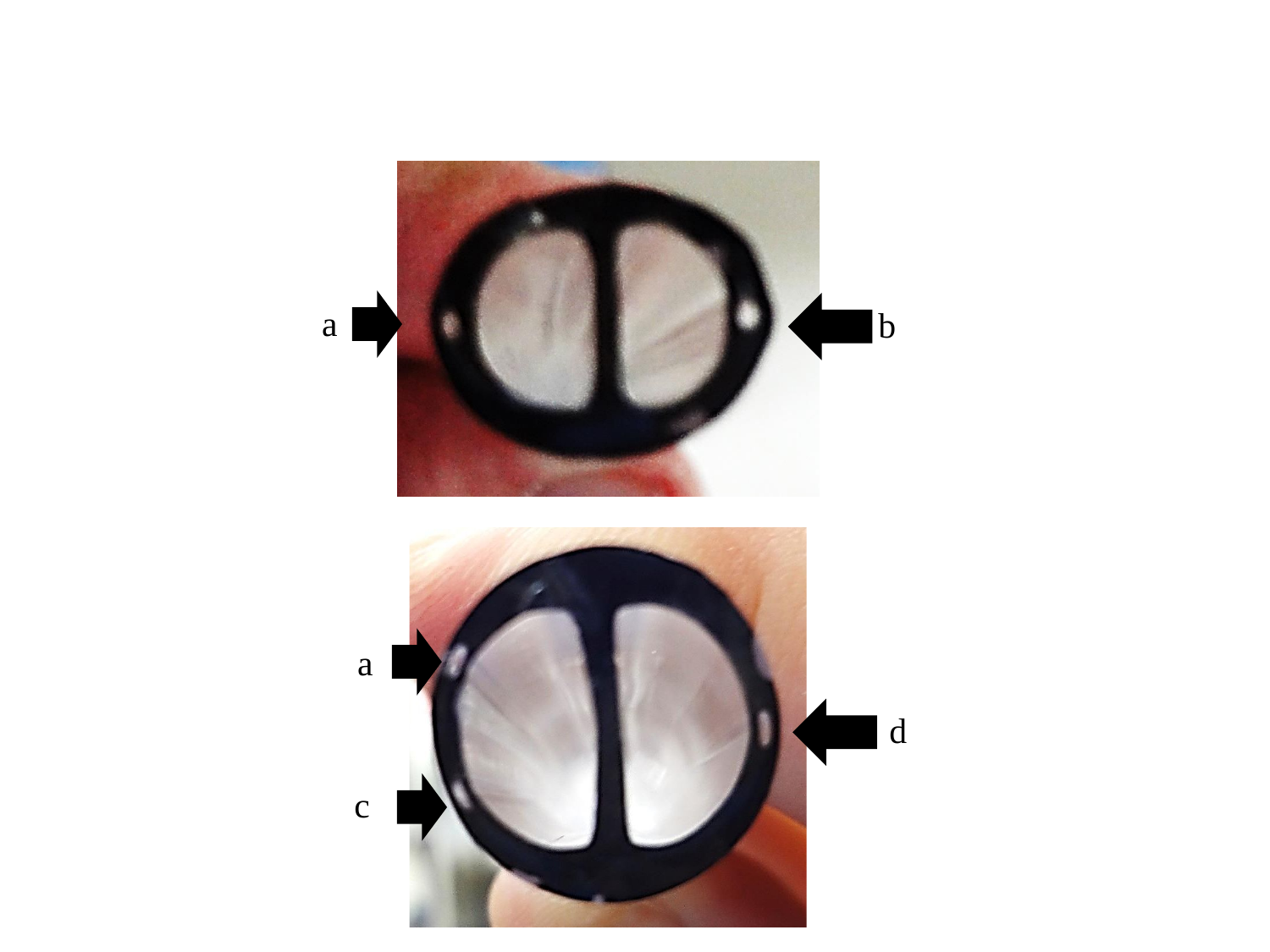

a
b
a
d
c

Supplement: Supplementary file 2 — Figure 2 Modified double-lumen tube. a Bronchial cuff lumen, b tracheal cuff lumen (X-ray opaque line), c tracheal cuff lumen, d no lumen (X-ray opaque line) (PPTX 1038 KB) [file 540_2018_2511_MOESM2_ESM.pptx]
